# Examining the Predictive Validity of the Grit Scale-Short (Grit-S) Using Domain-General and Domain-Specific Approaches With Student-Athletes

**DOI:** 10.3389/fpsyg.2022.837321

**Published:** 2022-05-03

**Authors:** James L. Rumbold, John G. H. Dunn, Peter Olusoga

**Affiliations:** ^1^Sport and Physical Activity Research Centre, Health Research Institute, Sheffield Hallam University, Sheffield, United Kingdom; ^2^Faculty of Kinesiology, Sport, and Recreation, University of Alberta, Edmonton, AB, Canada; ^3^Centre for Behavioural Science and Applied Psychology, Sheffield Hallam University, Sheffield, United Kingdom; ^4^Department of Public Health and Sport Sciences, Inland Norway University of Applied Sciences, Elverum, Norway

**Keywords:** burnout, dual career athletes, emotional exhaustion, perseverance, personality

## Abstract

This paper contributes to the debate as to whether grit is best conceptualized and measured as a domain-specific or domain-general construct. In the field of sport psychology, grit has traditionally been conceptualized and measured as a domain-general construct, with the majority of studies using the Grit Scale-Short (Grit-S: Duckworth and Quinn, 2009) to assess grit and its relationships with an array of personality-, performance-, and health-related outcomes. To date, no studies have compared the predictive validity of domain-general and domain-specific versions of the Grit-S with athletes who operate in different achievement settings. In a sample of United Kingdom student-athletes (*N* = 326, 214 males, 112 females; *M*age = 19.55 years, *SD* = 1.48 years), we examined the degree to which a domain-general version and two domain-specific versions of the Grit-S accounted for variance in two criterion variables that were either situated in an academic context (i.e., emotional exhaustion) or a sport context (i.e., competitive level). Results obtained from a series of hierarchical multiple regression analyses indicated that an academic-version of the Grit-S explained unique variance in academic emotional exhaustion beyond the variance explained by the domain-general version of the scale, and a sport-version of the Grit-S explained unique variance in competitive level beyond the variance explained by the domain-general version. Results support the adoption of domain-specific approaches to measure grit in specific achievement contexts. Our findings highlight the need for researchers to carefully consider the measurement approaches they adopt when studying grit in individuals who operate across different achievement settings.

## Introduction

Grit—which reflects an individual’s dispositional tendency to pursue goals over long periods of time despite adversity and setbacks (Duckworth et al., 2007)—has been identified as a personality characteristic that plays an important role in the achievement striving process in various performance domains. For example, higher levels of grit have been linked with higher attainment levels in academic settings (Credé et al., 2017), lower attrition rates in arduous military training/selection programs (Eskreis-Winkler et al., 2014), and higher performance levels in competitive sport (Cormier et al., 2021). Higher levels of grit have also been associated with better mental- and physical-health in students with chronic medical conditions (Traino et al., 2019) and reduced depression and suicidal ideation in military personnel (Pennings et al., 2015). As such, researchers typically regard grit as a personality characteristic that is adaptive for performance and health (Dunn et al., 2021).

Over the past 15 years, researchers who study grit have largely relied upon two instruments to measure the construct: the original 12-item Grit Scale (Grit-O; Duckworth et al., 2007) and the abbreviated 8-item Grit Scale-Short (Grit-S; Duckworth and Quinn, 2009). Both instruments contain two subscales labeled *perseverance of effort* (PE) and *consistency of interests* (CI). Although Duckworth and her colleagues (Duckworth et al., 2007; Duckworth and Quinn, 2009) originally conceptualized grit as a domain-general (i.e., global) personality disposition, Duckworth and Quinn did speak to the possibility that individuals may possess (or demonstrate) different levels of grit in different achievement settings.

Personality researchers have frequently acknowledged potential measurement benefits of adopting domain-specific personality inventories to enhance predictive validity in specific achievement settings (e.g., Lievens et al., 2008; Dunn et al., 2011; Credé, 2018). Lievens and colleagues argued that the reliability of contextualized personality inventories is often higher than that of domain-general measures. This increased reliability can be a function of reduced within-person variability of responses in contextually situated ‘frame-of-reference’ inventories because domain-general questionnaire items are often more open to interpretation by respondents than context-specific items (Lievens et al., 2008).

Recent studies in sport and performance psychology have provided evidence that grit may be best conceptualized and measured as a domain-specific construct. For example, using domain-general and domain-specific versions of the 12-item Grit-O, researchers (Cormier et al., 2019; Mosewich et al., 2021) reported that intercollegiate-level (varsity) student-athletes from a Canadian university had significantly higher mean PE and CI scores in sport than in academic and in non-contextualized general life settings (i.e., domain-general grit). These studies also highlighted that a domain-specific measure of academic grit explained significant amounts of unique variance in student Grade Point Average (GPA) beyond the variance explained by a domain-general measure. Similarly, in a study of high school students from Germany (Schmidt et al., 2019), an academic (i.e., domain-specific) measure of grit was a superior predictor of GPA than a domain-general measure of grit. Given these findings, more research investigating the potential benefits (and validity) of conceptualizing and measuring grit as a domain-specific construct is warranted.

Although studies assessing the predictive validity of the Grit-O have been valuable in establishing the importance of measuring grit as a domain-specific construct, a recent scoping review of 90 grit studies in sport psychology (Cormier et al., 2021) noted that the majority of research in sport (58%) has used the Grit-S (rather than the Grit-O). Moreover, when studies employ the Grit-S to measure grit in athletes, researchers have invariably chosen the domain-general version of the instrument (e.g., Atkinson and Martin, 2020; Albert et al., 2021; Howard et al., 2021; Doorley et al., 2022). Therefore, research examining the utility of the Grit-S as a domain-specific measure of grit is lacking, and the extent to which domain-specific versions of the Grit-S may enhance predictive validity for performance and health outcomes in specific achievement contexts is largely unknown.

A useful approach to establishing validity evidence that supports (or refutes) the domain-specific conceptualization of personality characteristics is to compare the degree to which domain-general and domain-specific instruments explain unique variance in theoretically-relevant criterion variables (Mosewich et al., 2021). If a domain-specific measure of grit cannot account for a significant amount of unique variance in a criterion variable *within the matched achievement domain of interest* beyond the variance accounted for by a domain-general measure, there would be little justification (i.e., validity evidence) to support a domain-specific approach. In contrast, incremental validity evidence supporting a domain-specific approach would be attained if a domain-specific measure of grit accounted for a significant amount of unique variance in a domain-relevant criterion variable beyond the variance accounted for by a domain-general measure. For example, support for a domain-specific approach would be obtained if (a) a measure of *academic grit* accounted for a significant amount of unique variance in a theoretically-relevant criterion variable (e.g., emotional exhaustion) in an *academic* setting beyond the variance explained by a domain-general measure of grit (Cormier et al., 2019), and/or (b) if a measure of *sport grit* accounted for unique variance in a theoretically-relevant criterion variable (e.g., competitive sport level) in a *sport* setting beyond the variance accounted for by a domain-general measure.

In the present study, we examined the extent to which the domain-general version of the Grit-S (Duckworth and Quinn, 2009) and two contextually-modified domain-specific versions of the Grit-S accounted for variance in student-athletes’ responses to two theoretically-relevant criterion variables. These criterion variables were either situated in an academic context or a sport context. The two criterion variables selected for this purpose were *academic emotional exhaustion* (a sub-dimension of academic burnout; Schaufeli et al., 2002), and *competitive sport level*. Previous research outside of sport settings has shown that higher levels of grit—whether treated as an overall (unitary) construct or multidimensional construct—tends to be associated with lower levels of emotional exhaustion and burnout (Cortez et al., 2020). In other words, grittier people tend to be less susceptible to experiencing burnout symptoms than less gritty people. To date, no studies have assessed how grit may explain student-athletes’ experiences of emotional exhaustion in academic contexts. This is important to examine given the variety of challenges and potential setbacks that student-athletes face when pursuing meaningful sport-, education-, and life-goals in their daily lives. Moreover, research highlights that student-athletes who experience burnout symptoms such as emotional exhaustion tend to experience heightened burnout symptoms over time (Sorkkila et al., 2018), which may ultimately result in drop out from sport or education entirely (Sorkkila et al., 2019). Examining the relationship between grit and academic emotional exhaustion in student-athletes is therefore important for researchers or practitioners who may be interested in developing grit interventions with the intention of enhancing well-being or preventing dropout.

Finally, previous research in the field of sport psychology has demonstrated that athletes who compete at higher (or more skilled) levels of competitive sport tend to have higher levels of grit than athletes who compete at lower (or less skilled) levels of sport (for a review see Cormier et al., 2021). Researchers have proposed that higher levels of grit help individuals sustain engagement in deliberate practice (Duckworth et al., 2007; Gilchrist et al., 2018) which, in turn, facilitates development and performance at higher levels of competition (Tedesqui and Young, 2017). Despite the existence of research evidence linking heightened grit with higher competitive levels (Tedesqui and Young, 2017), previous research assessing this relationship has utilized the Grit-O. Therefore, research is needed to determine whether the relationship between grit and competitive level holds when utilizing the more commonly adopted Grit-S with athletes.

The current study makes a unique contribution to the grit in sport literature by: (a) assessing domain-general *and* domain-specific versions of the Grit-S in a United Kingdom student-athlete sample; (b) assessing understudied performance attainment and health-related criterion variables, and (c) controlling for important demographic (sex) and academic (year of study) variables that have often been neglected in the grit literature. In accordance with Dunn and colleagues’ position that grit may be best measured as a domain-specific construct when the theoretically-relevant criterion variable of interest is regarded as a domain-specific construct (Dunn et al., 2021), we hypothesized that (a) a domain-specific measure of academic grit using the Grit-S would account for unique variance in academic emotional exhaustion beyond the variance accounted for by a domain-general version of the Grit-S, and (b) a domain-specific measure of sport grit using the Grit-S would account for unique variance in predicting competitive sport level beyond the variance accounted for by a domain-general version of the Grit-S.

## Materials and Methods

### Participants and Procedure

A total of 326 undergraduate students who were completing a degree in sport and exercise science at a university in the United Kingdom (214 males, 112 females) participated in the study. The age of the student-athletes ranged from 18 to 29 years (19.55 ± 1.48 years, mean ± SD). Of the total sample, 171 participants were in their first year of study, 80 were in their second year, and 75 were completing the final year of their 3-year degree. All participants indicated involvement in some level of competitive sport with 193 reporting that they competed at a recreational or club level and 133 reporting that they competed at a regional (e.g., county/state/intercollegiate) level or higher.

Institutional ethics approval was granted by the Sheffield Hallam University Faculty Research Ethics Committee (Ethics ID: HWB-2016-17-S&E-05). Participants were informed of the purpose of the study during classes held within the first two weeks of the academic year. At each information session (delivered in person by one of the research team), students were provided with a link to a Qualtrics online questionnaire, which also contained a participant information sheet and consent form to sign electronically. Students who wished to participate were given an opportunity to complete the survey at the start of the class and/or to complete the survey at a convenient time during the upcoming week. All participation was voluntary, students were free to participate (or decline participation) without consequence, and anonymity was assured. The Qualtrics online questionnaire contained questions focusing on demographic characteristics (age, sex, year of study, level of competitive sport engagement), dispositional grit, and academic emotional exhaustion.

### Measures

#### Grit

Grit was assessed by a domain-general version and two domain-specific versions of the Grit-S (Duckworth and Quinn, 2009). Following similar approaches to measuring grit employed in recent sport psychology research (Cormier et al., 2019; Mosewich et al., 2021), the domain-general version of the scale provided no situational frame of reference for participants to consider when responding to items (i.e., *general grit*). The stem for this scale was “this questionnaire contains 8 statements about perseverance in all aspects of your life”. In contrast, the two domain-specific versions directed participants to consider responses in the context of their academic studies (i.e., *academic grit*) or competitive sport involvement (i.e., *sport grit*). The stem for the academic grit scale was “please think about your answers in relation to your education. Think specifically about your approach to university and respond to the following items”. The stem for the sport grit scale was “please think about your answers in relation to your involvement in sport. Think specifically about training and competing and respond to the following items”. Each version of the Grit-S contains eight items; four items measure *perseverance of effort* (PE: e.g., “I am a hard worker”) and four items measure *consistency of interests* (CI: e.g., “I often set a goal but later choose to pursue a different one” [reverse score]). Participants rated items on a 5-point scale (1 = *not like me at all*; 5 = *very much like me*), with higher composite subscale scores (after reverse-scoring of CI items) reflecting higher levels of grit on each dimension.

The Grit-S has been used extensively in research to measure general grit in samples of athletes (e.g., Atkinson and Martin, 2020; Albert et al., 2021; Howard et al., 2021). However, to the best of our knowledge the Grit-S has not been used to measure domain-specific grit in academic and sport settings with student-athletes. For this reason, and because there is a high degree of inconsistency in terms of how sport psychology researchers report grit scores in the literature—with Grit-S scores either being presented as a single composite scale score or as separate subscale scores (Cormier et al., 2021)—we conducted preliminary psychometric analyses (see Results section) to assess the latent dimensionality/structure of each version of the scale. Determining the most appropriate way to work with Grit-S scores is important because “combining perseverance scores and consistency scores into an overall [composite] grit score…[can often] result in a significant loss in the ability to predict performance” (Credé et al., 2017, p. 502).

#### Emotional Exhaustion From Studies

Academic emotional exhaustion was measured by the emotional exhaustion subscale of the Maslach Burnout Inventory – Student Survey (Schaufeli et al., 2002). The subscale contains five items (e.g., “I feel emotionally drained by my studies”) that measure the degree to which students feel emotionally exhausted due to the demands of their academic studies. Participants rated items on a 7-point frequency scale (1 = *never*; 7 = *always*), with higher composite subscale scores reflecting higher levels of academic emotional exhaustion. Schaufeli et al. (2002) reported factorial validity evidence through good fitting confirmatory factor analysis models, acceptable levels of internal consistency (αs ≥ 0.74), and criterion-related validity evidence (in the form of significant negative correlations with variables measuring academic engagement) for the emotional exhaustion subscale in undergraduate samples. Internal consistency for the 5-item emotional exhaustion subscale in the current study was 0.81.

### Data Analysis

Missing data (< 5%) were replaced by computing an intra-individual mean-item score from the scores provided by the respondent on the remaining items in the corresponding subscale (Graham et al., 2003). To examine the latent dimensionality/structure of each version of the Grit-S we initially ran a series of maximum likelihood confirmatory factor analyses (CFA) upon the inter-item covariance matrices of each scale using LISREL (model 8.72, Scientific Software International, Inc., Lincolnwood, IL, United States). We tested a 1-factor model (with all items loading on a single grit factor) and a 2-factor model (with items loading, respectively, on PE or CI factors). Following the removal of one item from each version of the Grit-S (due to questionable factorial validity and internal reliability evidence), we conducted another set of CFAs on each 7-item version of the Grit-S and again examined 1- and 2-factor models. Finally, the latent dimensionality/structure of each Grit-S was examined using exploratory (i.e., Principal Component and Principal Axes) factor analyses.

Prior to conducting hierarchical multiple regression analyses, we screened the data for the presence of univariate and multivariate outliers (Tabachnick and Fidell, 2013). No univariate outliers were detected (i.e., all standardized z-scores were < |3.29|), however, three multivariate outliers were detected (i.e., Mahalanobis distances > 27.34, *p* < 0.001) and excluded from the regression analyses. We also screened for the presence of multicollinearity and influential data points. Across the regression analyses, all variance inflation factors (VIFs) were ≤ 3.47 and all Cook’s distances were ≤ 0.06, indicating that neither multicollinearity nor the presence of influential data points were a concern.

We conducted two hierarchical multiple regressions for each dependent variable (i.e., academic emotional exhaustion and competitive sport level) and altered the entry order of grit subscales across analyses. This served the purpose of examining the degree to which domain-general versus domain-specific versions of the Grit-S accounted for variance in the dependent variables. Given that grit levels have been shown to vary as a function of sex and year of study in university students (Kannangara et al., 2018), sex and year of study were entered as control variables in the first step of each analysis. In the first analysis to explain variance in academic emotional exhaustion, general PE and general CI were entered in the second step, followed by academic PE and academic CI in the third step. In the second analysis, the entry order of the grit scales was reversed, with academic grit entered in the second step and general grit entered in the third step. Given that emotional exhaustion was conceptualized as a domain-specific (i.e., academic) construct, sport grit was excluded from the two regression analyses used to predict emotional exhaustion.

Logistic regression analysis was conducted to explain the variance in competitive sport level as a function of sex, year of study, general grit, and sport grit. Adopting the same entry-order of independent variables described above, sex and year of study were entered as control variables in the first step of each analysis. In the first analysis, general PE and general CI were entered in the second step, and sport PE and sport CI were entered in the third step. In the second analysis, the entry order of the grit scales was reversed whereby sport grit subscales were entered in the second step and general grit subscales were entered in the third step. Given that competitive sport level represents a domain-specific criterion variable, academic grit was excluded from the logistic regression analyses.

## Results

### Psychometric Analyses of the Grit-S

Model fit indices (obtained from maximum likelihood CFAs) were superior for the 2-factor models than the respective 1-factor models (see Table 1) for each version of the Grit-S. Furthermore, all 2-factor models provided a significant improvement in model fit over each of the respective 1-factor models: general grit (χ^2^ [1] _difference_ = 157.98, *p* < 0.001; *w* = 0.70), academic grit (χ^2^ [1] _difference_ = 213.93, *p* < 0.001; *w* = 0.81), and sport grit (χ^2^ [1] _difference_ = 469.37, *p* < 0.001; *w* = 1.20). All of the standardized factor loadings for each version of the Grit-S were statistically significant, however, the absolute value of the factor loadings for Item 2 of the PE subscale (PE 0.2: “Setbacks don’t discourage me”) was < 0.30 for all three scales. Such a finding indicates that item PE 0.2 may be a questionable candidate for retention as a measure of perseverance of effort (in comparison to the other PE items) in the Grit-S. Moreover, when item PE 0.2 was removed from the PE subscale within each version of the Grit-S, internal consistency values (α) increased from 0.61 to 0.69 for general PE, 0.60 to 0.68 for academic PE, and 0.56 to 0.71 for sport PE. Given these findings, we removed item PE 0.2 from each PE subscale of the three Grit-S measures for all remaining analyses and re-ran the CFAs. The fit indices were marginal for the 7-item domain-general version of the Grit S (RMSEA = 0.09; CFI = 0.91; TLI/NNFI = 0.90, SRMR = 0.05), excellent for the 7-item Grit-S academic (RMSEA = 0.01; CFI = 0.1.0; TLI/NNFI = 1.0, SRMR = 0.03), but poor for the 7-item Grit-S sport (RMSEA = 0.15; CFI = 0.79; TLI/NNFI = 0.87, SRMR = 0.07).

**TABLE 1:** Fit indices following maximum likelihood confirmatory factor analyses for grit models.

	Model fit indices					
Model tested	χ^2^ (*df*), *p*	χ^2^/*df*	RMSEA [90% CI]	CFI	TLI/NNFI	SRMR
***General grit***						
1-factor	183.77 (20), *p* < 0.001	9.19	0.17 [0.15, 0.19]	0.74	0.64	0.10
2-factor	31.54 (19), *p* = 0.035	1.66	0.04 [0.01, 0.07]	0.98	0.97	0.05
***Academic grit***						
1-factor	267.68 (20), *p* < 0.001	13.38	0.21 [0.19, 0.23]	0.55	0.37	0.16
2-factor	53.76 (19), *p* < 0.001	2.83	0.07 [0.05, 0.10]	0.94	0.91	0.05
***Sport grit***						
1-factor	515.89 (20), *p* < 0.001	25.79	0.29 [0.27, 0.31]	0.43	0.21	0.20
2-factor	46.52 (19), *p* < 0.001	2.41	0.07 [0.04, 0.09]	0.97	0.95	0.05

*Inter-factor correlations in the 2-factor models were 0.49 (general grit), 0.12 (academic grit), and 0.31 (sport grit).*

Given the variability in model fit across the three scales following the removal of item PE 0.2, we further explored the suitability of working with 2-factor solutions for each version of the Grit-S. Rather than searching for better fitting models using modification indices (which greatly increases the likelihood of capitalizing on chance relationships within the data: MacCallum et al., 1992), we adopted the same procedures employed in studies that have previously examined the factor structure of domain-general and domain-specific versions of the 12-item Grit-O in samples of student-athletes where adequate fitting CFA models were not obtained (see Cormier et al., 2019; Mosewich et al., 2021). Specifically, we used exploratory factor analytic procedures and followed Velicer et al.’s (2000) directions by conducting Principal Component (PC) analyses on the correlation matrices for each 7-item version of the Grit-S, and determined the number of factors through examination of scree test and parallel analysis results. The scree plot for each version of the Grit-S clearly indicated the retention of two factors. Similarly, parallel analysis results indicated that only the first two eigenvalues for each PC solution (general grit [λ_1_ = 2.64, λ_2_ = 1.34, λ_3_ = 0.74]; academic grit [λ_1_ = 2.21, λ_2_ = 1.70, λ_3_ = 0.77]; sport grit [λ_1_ = 2.82, λ_2_ = 1.65, λ_3_ = 0.66]) were greater than the corresponding eigenvalues derived from the parallel analysis (λ_1_ = 1.21, λ_2_ = 1.12, and λ_3_ = 1.06). Given that scree and parallel analysis results converged upon the same number of factors, we retained two factors for all three versions of the Grit-S.

Following Cormier et al. (2019) and Mosewich et al. (2021), we then conducted Principal Axes factor analyses (with direct oblimin rotations [delta = 0]) on the inter-item correlation matrices for each of the 7-item versions of the Grit-S. The pattern matrices for each solution are shown in Table 2. Excellent simple structure (Thurstone, 1954) across all seven items in each solution was obtained; all pattern coefficients were ≥ 0.53 on the intended factor and ≤ | 0.14| on the non-intended factor. Given these results, we were confident that PE and CI should be treated as separate factors/subscales in all remaining analyses. Table 3 contains the internal consistency values for the PE and CI subscales of each version of the Grit-S following the removal of item PE 0.2.

**TABLE 2:** Pattern coefficients for principal axes factor analyses with direct oblimin rotations of the 7-item general, academic, and sport versions of the Grit-S.

			General		Academic		Sport	
	Item descriptions	Intended factor	*F1*	*F2*	*F1*	*F2*	*F1*	*F2*
1.	New ideas and projects sometimes distract me from previous ones. (R)	CI	**0.52**	–0.02	**0.53**	–0.04	**0.59**	0.01
3.	I have been obsessed with a certain idea or project for a short time, but later lost interest. (R)	CI	**0.61**	0.01	**0.67**	–0.01	**0.73**	–0.10
4.	I am a hard worker.	PE	–0.09	**0.71**	–0.08	**0.70**	0.00	**0.74**
5.	I often set a goal, but later choose to pursue a different one. (R)	CI	**0.66**	–0.05	**0.62**	0.01	**0.81**	–0.01
6.	I have difficulty maintaining my focus on projects that take more than a few months to complete. (R)	CI	**0.66**	0.17	**0.56**	0.04	**0.69**	0.18
7.	I finish whatever I begin.	PE	0.14	**0.56**	0.03	**0.59**	0.01	**0.70**
8.	I am diligent.	PE	0.02	**0.59**	0.05	**0.65**	–0.01	**0.61**

*Pattern coefficients > 0.30 are in boldface. Reverse-scored items are denoted by (R). Subscale abbreviations: CI, consistency of interests; PE, perseverance of effort. Inter-factor correlations were 0.40 (general grit), 0.17 (academic grit), and 0.29 (sport grit).*

**TABLE 3:** Means, standard deviations, correlations, and internal consistency values (α) for variables of interest.

	General grit		Academic grit		Sport grit		Emotional exhaustion	Sport level^a^	Sex^b^
Variables	PE	CI	PE	CI	PE	CI			
PE General	(0.69)	0.33***	0.76***	0.08	0.44***	0.05	−0.22***	0.11*	−0.13**
CI General		(0.71)	0.21***	0.50***	0.15**	0.45***	−0.30***	0.00	0.01
PE Academic			(0.68)	0.12*	0.38***	0.09	−0.26***	0.00	−0.15**
CI Academic				(0.68)	0.01	0.36***	−0.18**	−0.04	0.01
PE Sport					(0.71)	0.24***	−0.06	0.23***	0.05
CI Sport						(0.80)	−0.21***	0.01	0.02
Emotional exhaustion							(0.81)	−0.02	−0.09*
Sport level								−	−0.12*
*Mean*	3.86	3.07	3.88	3.03	4.23	3.37	3.93	−	−
*SD*	0.58	0.70	0.63	0.68	0.60	0.82	1.07	−	−

All statistics are computed using N, 326. Bivariate and biserial correlations are contained in the upper triangular matrix. Correlations between sex, sport level and all continuous variables were computed with Kendal’s τ. Internal consistency coefficients (α) are contained in parentheses in the main diagonal. PE, perseverance of effort; CI, consistency of interests. ^a^Sport level coded: 1, low, 2, high. ^b^Sex coded: 1, female, 2, male. *p < 0.05; **p < 0.01; ***p < 0.001.

### Descriptive Statistics

The means, standard deviations, and bivariate correlations among the grit and emotional exhaustion variables are contained in Table 3. Table 3 also contains biserial (Kendall’s Tau) correlations between each of these continuous variables and competitive sport level (coded: 1 = low level, 2 = high level) and sex (coded: 1 = female, 2 = male).

### Examining Incremental Validity of Domain-Specific Versions of the Grit-S

#### Emotional Exhaustion From Studies

Results of the two hierarchical regression analyses to explain variance in academic emotional exhaustion are shown in Table 4. In the first analysis, sex and year of study (Step 1) accounted for 3% of the variance (*p* < 0.01) in emotional exhaustion. Year of study primarily contributed to the effect (β = 0.14, *p* < 0.05); students who were further into their academic programs tended to report higher levels of academic emotional exhaustion. General grit subscales (Step 2) accounted for an additional 11% of unique variance (*p* < 0.001) in academic emotional exhaustion beyond sex and year of study. Both general PE (β = −0.16, *p* < 0.01) and general CI (β = −0.24, *p* < 0.001) were inversely associated with emotional exhaustion. The inclusion of the academic grit subscales in Step 3 accounted for an additional 2% of unique variance (*p* < 0.05) in emotional exhaustion beyond sex, year of study, and general grit; academic PE (β = −0.24, *p* < 0.01) primarily contributed to the effect.

**TABLE 4:** Two multiple regression analyses predicting emotional exhaustion in academic studies.

First regression analysis							Second “Reverse Entry Order” regression analysis						
	Predictor	*R* ^2^	Δ*R*^2^	Δ*F*	β	*T*		Predictor	*R* ^2^	Δ*R*^2^	Δ*F*	β	*T*
Step 1		0.03	0.03	4.87**			Step 1		0.03	0.03	4.87**		
	Sex				–0.11	–1.93		Sex				–0.11	–1.93
	Year of study				0.14	2.56*		Year of study				0.14	2.56*
Step 2		0.14	0.11	20.45***			Step 2		0.13	0.10	17.86***		
	Sex				–0.13	−2.39*		Sex				–0.15	−2.80**
	Year of study				0.12	2.33*		Year of study				0.15	2.81**
	PE General				–0.16	−2.81**		PE Academic				–0.26	−4.74***
	CI General				–0.24	−4.28***		CI Academic				–0.16	−2.93**
Step 3		0.16	0.02	4.38*			Step 3		0.16	0.03	6.76**		
	Sex				–0.14	−2.62**		Sex				–0.14	−2.62**
	Year of study				0.13	2.39*		Year of study				0.13	2.39*
	PE General				0.03	0.27		PE Academic				–0.24	−2.68**
	CI General				–0.23	−3.49***		CI Academic				–0.04	–0.68
	PE Academic				–0.24	−2.68**		PE General				0.03	0.27
	CI Academic				–0.04	–0.68		CI General				–0.23	−3.49***

Subscale abbreviations: PE, perseverance of effort; CI, consistency of interests. *p < 0.05; **p < 0.01; ***p < 0.001.

In the second analysis where the entry order of the grit scales was reversed (see Table 4), academic grit subscales accounted for 10% of the variance (*p* < 0.001) in emotional exhaustion beyond sex and year of study. Both academic PE (β = −0.26, *p* < 0.001) and academic CI (β = −0.16, *p* < 0.01) contributed to the effect. The negative regression coefficients indicate that student-athletes reporting higher academic PE and academic CI tended to report lower academic emotional exhaustion. The inclusion of the general grit subscales in Step 3 accounted for an additional 3% of unique variance (*p* < 0.01). General CI primarily contributed to the effect (β = −0.23, *p* < 0.001).

#### Competitive Sport Level

A test of the full model with all six predictor variables was statistically significant (χ^2^ [6, *N* = 326] = 25.84, *p* < 0.001), indicating that the set of predictor variables significantly distinguished between low- and high-level competitive sport performers. In the first logistic regression analysis, sex and year of study (Step 1) accounted for 2% of the variance in competitive sport level (see Table 5). Sex was an inverse predictor of competitive sport level, such that females were more likely to report competing at a higher sport level than males (β = −0.51, *p* < 0.05). However, the odds ratio showed little change in the likelihood of female student-athletes competing at a higher level than male student-athletes. General grit (Step 2) failed to explain the probability of competing at a higher or lower level of sport. The inclusion of sport grit subscales in Step 3 accounted for an additional 6% of unique variance (*p* < 0.001) in competitive sport level. Sport PE significantly predicted competitive sport level, indicating that student-athletes who displayed higher sport PE had a greater likelihood of participating at a higher competitive sport level (β = 0.95, *p* < 0.001). Moreover, the odds ratio supports the likelihood of competing at a higher sport level, based on a two-unit increase in Sport PE. In the second analysis where the entry order of grit scales was reversed (see Table 5), sport grit (Step 2) accounted for 8% of the variance in competitive sport level beyond sex and year of study; sport PE (β = 0.93, *p* < 0.001) primarily contributed to the effect with an odds ratio of 2.52. The inclusion of the domain-general grit dimensions in Step 3 failed to account for a significant amount of unique variance in competitive sport level beyond sex, year of study, and sport grit. Classification to competitive sport level revealed that 81 of low- and 38% of high-level student-athletes were correctly predicted, with an overall success rate of 63%.

**TABLE 5:** Two logistic regression analyses predicting the likelihood of competitive sport level.

First logistic regression analysis							Second “Reverse Entry Order” logistic regression analysis						
		*R* ^2^	Δ*R*^2^	β	*Wald*	OR (95% CI)			*R* ^2^	Δ*R*^2^	β	*Wald*	OR (95% CI)
Step 1		0.02	0.02				Step 1		0.02	0.02			
	Sex			0.51*	4.67	0.60 (0.38, 0.95)		Sex			−0.51*	4.67	0.60 (0.38, 0.95)
	Year of study			–0.12	0.71	0.89 (0.68, 1.17)		Year of study			–0.12	0.71	0.89 (0.68, 1.17)
Step 2		0.04	0.02				Step2		0.10	0.08			
	Sex			−0.44^†^	3.25	0.65 (0.40, 1.04)		Sex			−0.62**	6.32	0.54 (0.33, 0.87)
	Year of study			–0.15	1.10	0.86 (0.65, 1.14)		Year of study			–0.09	0.42	0.91 (0.69, 1.21)
	PE General			0.41^†^	3.62	1.51 (0.99, 2.30)		PE Sport			0.93***	18.08	2.52 (1.65, 3.87)
	CI General			–0.14	0.59	0.87 (0.62, 1.24)		CI Sport			–0.12	0.68	0.89 (0.67, 1.18)
Step 3		0.10	0.06				Step3		0.10	0.00			
	Sex			−0.63**	6.20	0.53 (0.32, 0.87)		Sex			−0.63**	6.20	0.53 (0.32, 0.87)
	Year of study			–0.10	0.45	0.91 (0.68, 1.21)		Year of study			–0.10	0.45	0.91 (0.68, 1.21)
	PE General			–0.05	0.03	0.96 (0.58, 1.57)		PE Sport			0.95***	14.85	2.58 (1.59, 4.18)
	CI General			–0.07	0.10	0.94 (0.62, 1.42)		CI Sport			–0.10	0.33	0.91 (0.65, 1.26)
	PE Sport			0.95***	14.85	2.58 (1.59, 4.18)		PE General			–0.05	0.03	0.96 (0.58, 1.57)
	CI Sport			–0.10	0.33	0.91 (0.65, 1.26)		CI General			–0.07	0.10	0.94 (0.62, 1.42)

*Subscale abbreviations: PE, perseverance of effort; CI, consistency of interests.*

^†^p ≤ 0.07; *p < 0.05; **p < 0.01; ***p < 0.001.

## Discussion

The purpose of this study was to establish validity evidence that either supported or refuted the domain-specific assessment of grit using the Grit-S in achievement settings with student-athletes. We examined the predictive utility of using a domain-general and two domain-specific versions of the Grit-S (Duckworth and Quinn, 2009) to explain variance in theoretically relevant context-specific criterion variables. We hypothesized that (a) an academic version of the Grit-S would account for unique variance in academic emotional exhaustion beyond the variance explained by a domain-general version of the scale, and (b) a sport version of the Grit-S would account for unique variance in competitive sport level beyond the variance explained by a domain-general version of the scale. Overall, regression results supported these hypotheses.

Results of regression analyses that examined whether domain-specific grit accounted for unique variance in academic emotional exhaustion (Table 4) and competitive sport level (Table 5) beyond domain-general grit supported the assessment of grit as a domain-specific construct in specific achievement settings. Academic grit subscales explained unique variance (2%) in academic emotional exhaustion beyond the variance accounted for by sex, year of study, and general grit (Table 4). Sport grit subscales accounted for a significant amount of unique variance (6%) in competitive sport level beyond the variance accounted for by sex, year of study, and general grit (Table 5). These are important findings because, to the best of our knowledge, previous research using the Grit-S with athletes has exclusively conceptualized and measured grit as a domain-general construct. It appears that a greater understanding of grit in specific achievement settings can be attained when grit is conceptualized and measured as a domain-specific construct.

Although we argue that a domain-specific approach is warranted when measuring grit in specific achievement settings, we note that the domain-general version of the Grit-S accounted for unique variance in academic emotional exhaustion (Table 4) regardless of whether the domain-general version of the scale was entered in the regression analyses before or after the academic version of the scale. Such a finding supports the *combined* assessment of grit using both domain-general and domain-specific approaches. Regardless of the entry-order of the grit scales in the regression analyses, academic PE and general CI were both inversely associated with academic emotional exhaustion. Such a finding confirms previous research indicating that higher levels of grit are often associated with lower levels of emotional exhaustion and burnout (Mullen and Crowe, 2018; Cortez et al., 2020). To our knowledge, this is the first study in sport to assess the relationship between any measure of grit and academic emotional exhaustion in student-athletes.

When examining how general grit and sport grit accounted for the likelihood of student-athletes competing at a higher or lower level of sport (Table 5), domain-general grit did not significantly predict membership to a higher competitive level (*p* ≤ 0.07), whereas sport grit did (regardless of the entry order in the logistical regression analyses). These results provide support for a domain-specific approach to measuring grit in sport. The fact that higher sport PE was associated with a higher probability of competing at a higher level of sport is also in line with previous research which illustrates that athletes who compete in higher (or more skilled) levels of sport tend to have higher levels of grit than athletes who compete in lower (or less skilled) levels of sport (Cormier et al., 2021).

Collectively, the regression results (Tables 4, 5) provide incremental validity evidence that domain-specific measures of grit can explain unique variance in theoretically relevant domain-matched criterion variables beyond the variance explained by domain-general measures of grit. In line with the work of Lievens et al. (2008) on ‘frame-of-reference’ personality instruments, it is conceivable that the domain-specific versions of the Grit-S reduce “within-person inconsistency” (p. 277) of participant responses to items (in comparison to responses on domain-general measures), which in turn reduces measurement error and improves validity. Regardless of why the domain-specific measures of grit showed evidence of incremental validity, our findings lend support to a growing body of research evidence that points to the potential benefits of measuring grit as a domain-specific construct in achievement contexts (Clark and Malecki, 2019; Cormier et al., 2019; Schmidt et al., 2019; Mondak, 2020; Mosewich et al., 2021). We suggest that sport and performance psychology researchers who wish to identify associations between grit dimensions and theoretically relevant criterion variables in specific achievement domains will likely benefit from adopting a domain-specific approach to measuring grit. We call for more research that examines different theoretically relevant criterion variables (than those used in the current study) across different achievement settings to examine the validity of our recommendation.

Building upon our discussion of how sport psychology researchers measure grit, it is important to consider the implications of the current factor analytic results surrounding the latent dimensionality (and factor structure) of each version of the Grit-S. Model-fit indices (Table 1) and EFA results (in terms of scree plot and parallel analysis results) clearly indicated that the latent dimensionality of all three versions of the Grit-S was best captured when PE and CI items loaded on separate factors as opposed to loading on a single grit factor. Indeed, the degree to which simple structure was exhibited across all items in each EFA solution (Table 2) appears to reinforce the appropriateness of treating PE and CI as separate constructs. Collectively, these results support the position of Tynan (2021) who recently argued that there “is growing evidence that perseverance [of effort] and consistency [of interests] are two separate constructs, and ‘overall grit’ is not psychometrically meaningful” (p. 144). These results make an important contribution of clarity because there is a high degree of inconsistency in the sport psychology literature with respect to whether researchers work with a single composite grit score (which is akin to treating grit as a unidimensional construct) or separate PE and CI subscale scores (which treats grit as a multidimensional construct) (Cormier et al., 2021). Credé and his colleagues addressed this issue in a series of meta-analysis and commentary papers outside the field of sport psychology (Credé et al., 2017; Credé, 2018, 2019) and questioned the validity of combining PE and CI subscales into a single composite score (using either Grit-O or Grit-S scores). Guo et al. (2019) reinforced this position stating that the aggregation of “CI and PE into a single construct is not empirically justifiable” (p. 3938). We encourage researchers in the field of sport psychology to examine the practical and theoretical implications of treating PE and CI as separate constructs versus combining them into a single grit construct. On the basis of our findings, we caution researchers against working with composite (i.e., unitary) grit scores unless compelling empirical and theoretical evidence to do so is presented.

### Limitations

The current study contains a number of limitations that require attention in future research. First the cross-sectional design limits the degree to which causal inferences can be generated. For example, on the basis of our findings, we cannot determine if higher academic grit leads to a reduction in academic emotional exhaustion or if higher emotional exhaustion in academic studies causes reductions in academic grit, nor can we determine if higher grit in sport enables athletes to compete at higher levels of sport, or if athletes who compete at higher levels develop higher grit in order to deal with the increased training and competitive demands inherent within high-level sport. Future research using prospective and longitudinal designs to address these issues are required.

We acknowledge that the internal consistency of the general PE, academic PE, and academic CI subscales was marginal (with αs ranging from 0.66 to 0.68). Some degree of caution must therefore be used when making inferences based upon results associated with these subscales. Moreover, we note that a number of studies that have used the domain-general version of the Grit-S with athletes have also reported internal consistency values < 0.70 at the subscale level (e.g., Light Shields et al., 2018; Newland et al., 2020) and/or composite scale level (e.g., Larkin et al., 2016; Albert et al., 2021). Researchers may attempt to alleviate some of these psychometric ‘internal-consistency issues’ by adopting the longer Grit-O (which contains six items per subscale) rather than the Grit-S given that the value of coefficient alpha is mathematically dependent upon the number of items within a scale or subscale. We further note that Duckworth et al. (2021) recently acknowledged that saving test administration time with the 8-item Grit-S may not be “worth the cost of omitting four Grit Scale [i.e., Grit-O] items with strong content validity” (p. 574). Such sentiment further points to the potential benefits of using the Grit-O (over the Grit-S) as the instrument of choice when measuring grit in athletes (for a related discussion see Tynan, 2021).

Another potential limitation of this study relates to the dichotomous assessment of competitive sport level that we employed. A more nuanced assessment of this variable (with more refined distinctions between different competitive levels) might lead to greater variability in sport-level scores and impact the magnitude of the relationships between sport level and grit that were observed in this study. As noted previously, we posit that future research examining the potential benefits of adopting a domain-specific approach (over a domain-general approach) to measuring grit in sport would likely benefit from using other domain-matched criterion variables (beyond competitive level) that are expected to be theoretically linked to grit in sport. Such criterion variables might include athletic burnout (Howard et al., 2021), time spent by athletes engaged in training (i.e., deliberate practice) to enhance sport performance (Larkin et al., 2016), and/or domain-specific individual-difference characteristics that operate in sport such as mental toughness (Fawver et al., 2020), self-compassion (Mosewich et al., 2021) and perfectionism (Dunn et al., 2021).

Our decision to focus solely upon emotional exhaustion limits the degree to which our findings generalize to other dimensions of burnout that can exist in academic settings (e.g., cynicism and professional efficacy; Schaufeli et al., 2002). Indeed, future research might benefit from assessing relationships between grit and various dimensions of burnout (e.g., reduced sense of accomplishment, physical/emotional exhaustion, and activity devaluation) to determine the extent to which heightened grit might act as a protective factor against burnout resulting from exposure to environmental (or workload) demands in different achievement settings (Teuber et al., 2021). Finally, we acknowledge that this study employed a sample of student-athletes who were studying for a 3-year undergraduate degree at a single university in the United Kingdom. It is possible that results might have differed had we sampled student-athletes who were taking 4- or 5-year degree programs (as is common in programs undertaken in other countries worldwide). Similarly, our results might have differed had we worked with student-athletes competing at higher or lower levels of sport. It also remains to be determined whether the current results are generalizable to younger student-athletes who are studying in high school and who may be competing in youth sport settings.

## Conclusion

Given the rising frequency of grit research in sport over the last 7 years (Cormier et al., 2021) and the position held by many researchers that heightened grit has achievement-striving benefits (Tedesqui and Young, 2017; Gilchrist et al., 2018; Dunn et al., 2021) and health benefits (Pennings et al., 2015; Traino et al., 2019; Cortez et al., 2020), we recommend that researchers carefully consider the measurement approaches they adopt when studying grit in athletes. Our findings make an important contribution to clarify international discussion on the dimension structure and measurement of grit. Namely, grit should no longer be measured as a unitary construct, but rather, one consisting of two separate but interrelated constructs (perseverance of effort; consistency of interests), that should also be measured in contextually specific ways. Although results of this study support the adoption of domain-specific approaches to measuring grit in achievement settings, we also reiterate the position of Dunn and colleagues that researchers should not abandon the domain-general assessment of grit but should instead seek to determine “when, where, and under what conditions it may be more valuable to use a domain-specific approach” (Dunn et al., 2021, p. 218).

## Data Availability Statement

The raw data supporting the conclusions of this article will be made available by the authors, without undue reservation.

## Ethics Statement

The studies involving human participants were reviewed and approved by Faculty of Health and Wellbeing Research Ethics Committee (Ethics ID: HWB-2016-17-S&E-05), Sheffield Hallam University. The patients/participants provided their written informed consent to participate in this study.

## Author Contributions

JR and PO: conceptualization and investigation. JR and JD: statistical analysis and writing – original draft and edited manuscript. All authors have read and approved the final version of the manuscript, and agreed with the order of presentation of the authors.

## Conflict of Interest

The authors declare that the research was conducted in the absence of any commercial or financial relationships that could be construed as a potential conflict of interest.

## Publisher’s Note

All claims expressed in this article are solely those of the authors and do not necessarily represent those of their affiliated organizations, or those of the publisher, the editors and the reviewers. Any product that may be evaluated in this article, or claim that may be made by its manufacturer, is not guaranteed or endorsed by the publisher.
